# Trends in the prevalence of congenital hydrocephalus in 14 cities in Liaoning province, China from 2006 to 2015 in a population-based birth defect registry from the Liaoning Women and Children’s Health Hospital

**DOI:** 10.18632/oncotarget.24239

**Published:** 2018-01-13

**Authors:** Yan-Hong Huang, Qi-Jun Wu, Yan-Ling Chen, Cheng-Zhi Jiang, Ting-Ting Gong, Jing Li, Li-Li Li, Chen Zhou

**Affiliations:** ^1^ Department of Science and Education, Shenyang Maternity and Child Health Hospital, Shenyang, China; ^2^ Department of Clinical Epidemiology, Shengjing Hospital of China Medical University, Shenyang, China; ^3^ Liaoning Women and Children's Health Hospital, Shenyang, China; ^4^ School of Environmental and Chemical Engineering, Shenyang Ligong University, Shenyang, China; ^5^ Department of Obstetrics and Gynecology, Shengjing Hospital of China Medical University, Shenyang, China; ^6^ Department of Children's Health Prevention, Shenyang Maternity and Child Health Hospital, Shenyang, China; ^7^ Department of Information Statistics, Shenyang Maternity and Child Health Hospital, Shenyang, China

**Keywords:** congenital hydrocephalus, Liaoning province, poisson regression, prevalence, time trend

## Abstract

The aim of this study was to assess the prevalence and trends of congenital hydrocephalus (CH) using a large population-based sample with cases retrieved from the Liaoning Birth Defects Registry, which included 14 cities, over a 10-year period. CH prevalence, percent change, average change, and contribution rates of each city were calculated. Statistical analysis was performed using a Poisson regression model. There was a total of 3008 CH cases among 3,248,954 live births during the observational period (9.26 cases/10,000 live births). On average, the prevalence of CH had significantly decreased by 11.84% each year (*P* < 0.01). The three leading cities with the highest prevalence of CH were Chaoyang (13.73/10,000), Huludao (13.18/10,000), and Fuxin (12.71/10,000), while Yingkou (6.06/10,000), Dalian (6.27/10,000), and Anshan (6.56/10,000) had the lowest prevalence. Notably, significantly decreasing trends were observed in 10 (71.4%) of these 14 cities. In addition, the cities of Fushun, Shenyang, and Dalian had contributed to more than one-third of the decreasing trend in Liaoning province. In conclusion, this study provides evidence of the decreasing prevalence of CH over a 10-year period (2006–2015) in Liaoning province. Preventative efforts should be strengthened to further reduce the risk of CH in these high prevalence areas.

## INTRODUCTION

According to the International Classification of Diseases (10th Revision) published by the World Health Organization, congenital hydrocephalus (CH) is characterized by an abnormal accumulation of cerebrospinal fluid (CSF) within the cerebral ventricles, which results from an imbalance between production and absorption [1]. Around 18−20 weeks of gestation, the first prenatal signs of hydrocephalus may be visible on ultrasound as the ‘‘banana sign’’, however, in some cases the hydrocephalus is only visible later in gestation. Although the accumulation of CSF in the majority of CH cases is reduced through the widespread use of CSF shunting, the fetus or infant often faces multiple surgical procedures resulting in significant morbidity [1, 2], which continues to be a worldwide public health burden. Obstruction of the cerebral aqueduct flow, Arnold-Chiari malformation, and Dandy-Walker malformation are the most common causes of CH [3]. Hydrocephalus is categorized as congenital or acquired; however, it is often difficult to distinguish between these entities in neonates because CH at birth may be secondary to another pathology [4]. In addition, hydrocephalus present at birth can remain subclinical until aging or trauma causes the disease to become symptomatic [4–7]. Therefore, the prevalence of CH remains poorly defined, not only because of the various definitions of CH between studies, but also the limited number of studies describing the differences between congenital and acquired hydrocephalus.

The prevalence of CH across time and regions ranges from 4.7 to 136.9 cases per 10,000 live births [8–18]. For example, Garne et al [9] observed eighty-seven congenital hydrocephalus cases (4.65 per 10,000 livebirths) during the period 1996–2003 from four European registries of congenital malformations (EUROCAT). For comparison, Fernell et al [18] found 13.69 per 1000 very preterm infants covering the birth years 1991–1994 in western Sweden. Furthermore, some studies have observed an increased prevalence of CH between the 1960s and 1990s, which might be attributed to the increased survival of extremely preterm infants [18, 19]. In contrast, several subsequent studies have suggested that the prevalence of CH has decreased in recent years [10, 13, 18]. This phenomenon has been thought to result from improved care of extremely preterm infants and the advent of folic acid supplementation or food fortification as well as the increasing rate of fetal termination after prenatal diagnosis of CH [20]. As compared to other countries, few studies have described the trends in the prevalence of CH in China, regardless of the observational period. For example, Wang et al [21] suggested that the prevalence of CH was 8.28 cases per 10,000 live births in China from 1988 to 1992. In addition, a decreasing trend was observed during this period [21]. In contrast, Dai et al. [8] reported 3012 perinatal cases of CH among 4,282,536 births in China from 1996 to 2004, yielding a prevalence of 7.03 cases per 10,000 live births. Notably, the annual prevalence of CH tended to increase during that period. However, the prevalence of CH over the past decade and whether a similar decreasing trend could still be observed in other cities remains unknown. As one of the most important provinces in China, Liaoning province covers an area of 145,900 km^2^ with a population of almost 42 million, which has greatly contributed to the development of China over the past few decades. Nevertheless, there has been no formal assessment of the prevalence of CH in this population. Therefore, the aim of the present study was to determine the prevalence of CH among infants in Liaoning province over a 10-year period from 2006 to 2015.

## RESULTS

The numbers of live births in the 14 cities in Liaoning province during the 10-year observational period are presented in Table 1. During this period, the overall number of live births was highest in 2014 (364,400) and lowest in 2015 (298, 437). In addition, when compared by city, Shenyang, the capital city of Liaoning province, had the largest number of live births in each year and Benxi had the smallest.

**Table 1:** The number of live births in each city in Liaoning province, 2006 to 2015

City	Year										Overall
	2006	2007	2008	2009	2010	2011	2012	2013	2014	2015	
Liaoning Province	306,734	341,432	330,414	321,353	307,826	304,079	353,108	321,171	364,400	298,437	3,248,954
Shenyang	52,256	61,108	59,196	59,200	57,521	58,335	69,721	67,854	80,997	65,118	631,306
Dalian	38,744	46,652	48,309	47,900	48,774	50,490	62,324	58,722	71,178	57,641	530,734
Anshan	29,270	31,305	29,647	27,721	25,184	25,603	28,790	25,855	36,171	20,798	280,344
Fushun	11,661	12,997	12,314	12,337	11,638	11,556	12,942	12,016	12,845	10,138	120,444
Benxi	8620	9435	8759	8842	8696	8261	9440	8700	9857	7627	88,237
Dandong	15,710	15,725	14,836	14,274	13,894	14,038	15,895	15,111	17,718	14,278	151,479
Jinzhou	24,293	24,261	23,149	22,342	21,255	20,098	22,559	20,860	16,137	16,985	211,939
Yingkou	16,987	18,924	19,667	19,070	17,947	18,484	21,309	14,224	21,684	16,515	184,811
Fuxin	14,158	14,142	13,353	13,322	12,370	11,800	13,050	9662	9121	11,752	122,730
Liaoyang	12,888	15,039	13,754	13,200	12,331	11,386	13,296	11,702	12,747	9251	125,594
Panjing	9887	9669	10,134	9009	8800	8867	10,362	9644	8276	9197	93,845
Tieling	21,263	20,298	21,456	19,854	18,421	16,945	18,938	14,960	17,389	15,269	184,793
Chaoyang	28,669	30,980	31,168	30,574	27,837	27,207	31,236	29,919	30,646	26,083	294,319
Huludao	22,328	30,897	24,672	23,708	23,158	21,009	23,246	21,942	19,634	17,785	228,379

**Figure 1:**
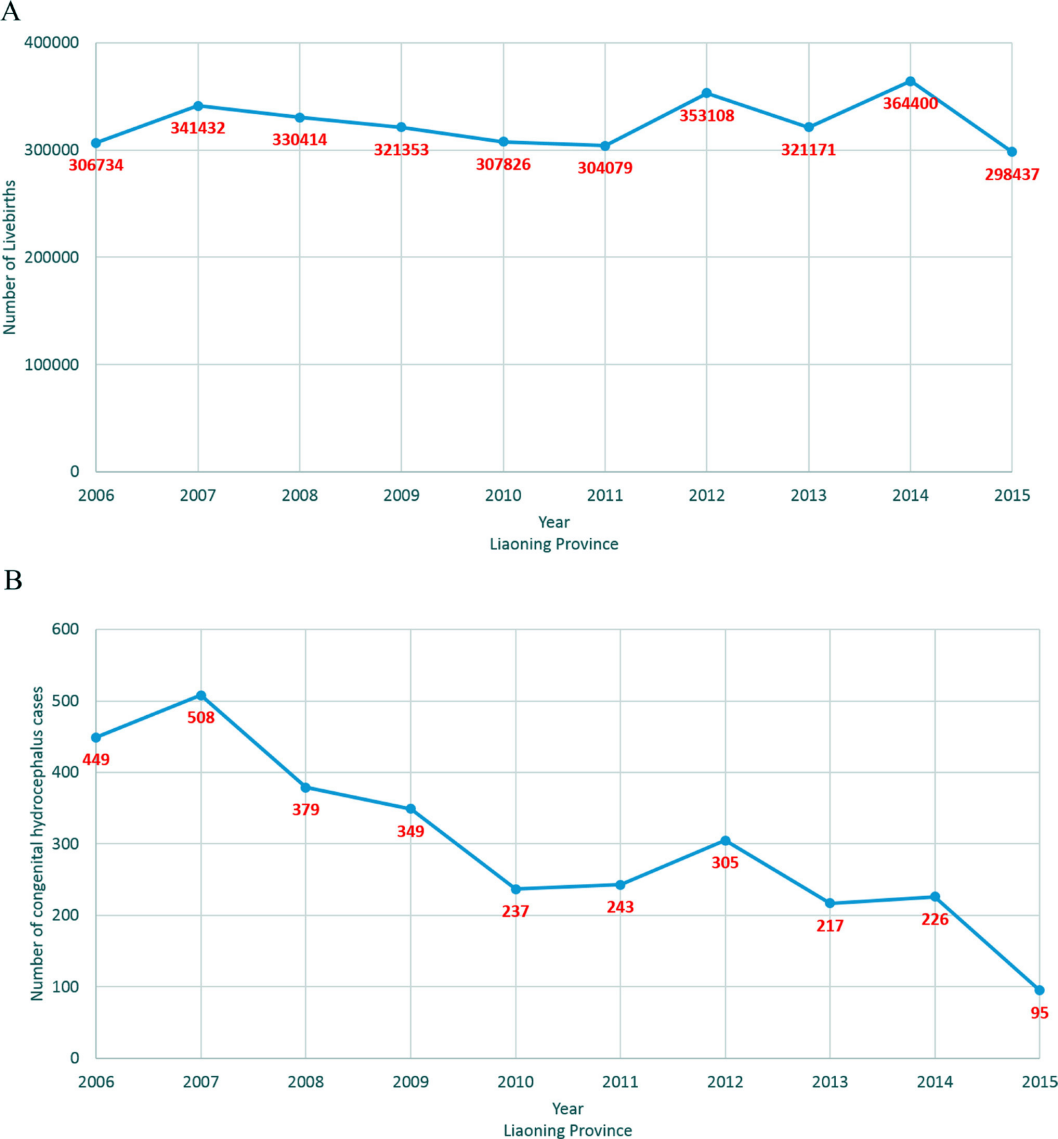
Number of newborns and congenital hydrocephalus cases from 2006 to 2015 on the basis of a population-based birth defect registry from Liaoning women and children's health hospital (**A**) newborns; (**B**) congenital hydrocephalus.

**Figure 2:**
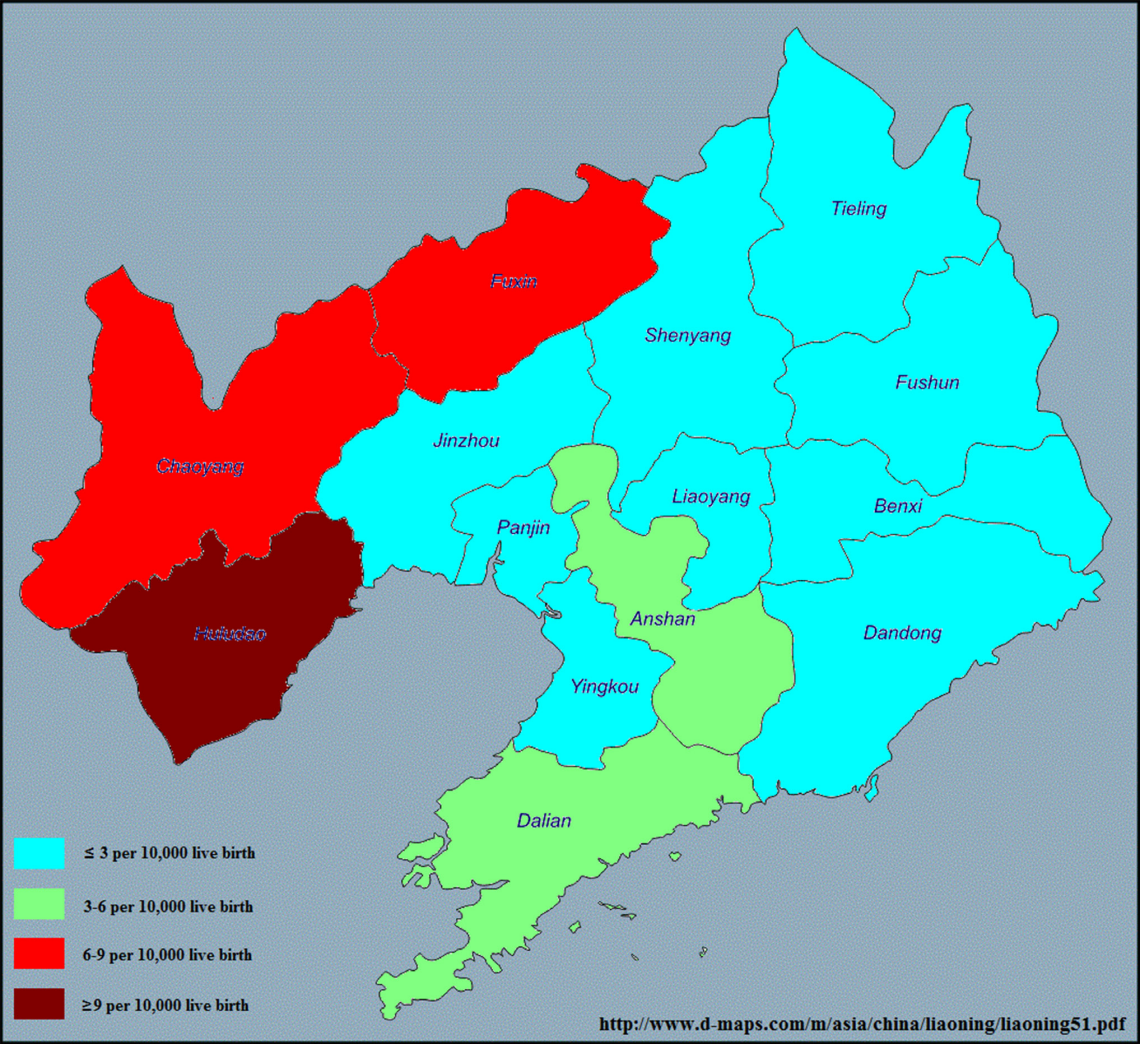
Prevalence of congenital hydrocephalus in each of 14 cities in Liaoning province from 2006 to 2015 on the basis of a population-based birth defect registry from Liaoning Women and Children's Health Hospital www.d-maps.com/m/asia/china/liaoning/liaoning51.pdf

The prevalence of CH in each city in Liaoning province is demonstrated in Figure 1 and Figure 2. During the 10-year period of 2006–2015, 3008 cases of CH were detected among 3, 248, 954 live births (prevalence of 9.26 cases per 10,000 live births). Chaoyang (13.73 per 10,000 live births), Huludao (13.18 per 10,000 live births), and Fuxin (12.71 per 10,000 live births) recorded the highest rates of CH in Liaoning province. In contrast, Yingkou (6.06 per 10,000 live births), Dalian (6.27 per 10,000 live births), and Anshan (6.56 per 10,000 live births) recorded the lowest rates of CH.

**Figure 3:**
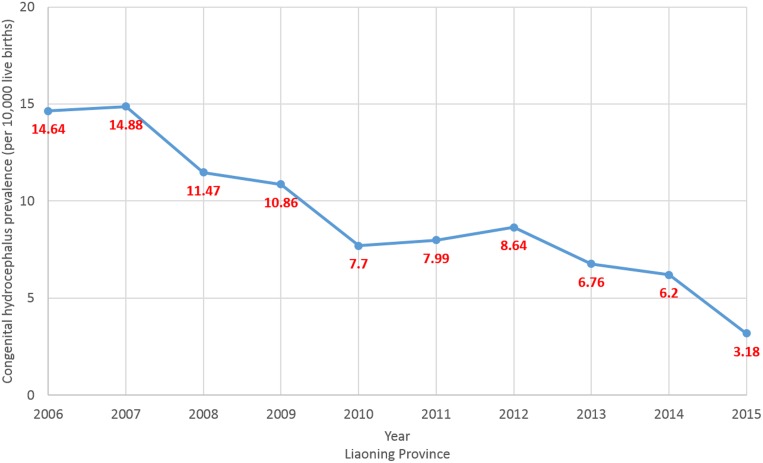
Trends in congenital hydrocephalus prevalence (per 10,000 live births) in Liaoning province from 2006 to 2015 on the basis of a population-based birth defect registry from Liaoning Women and Children's Health Hospital

**Figure 4:**
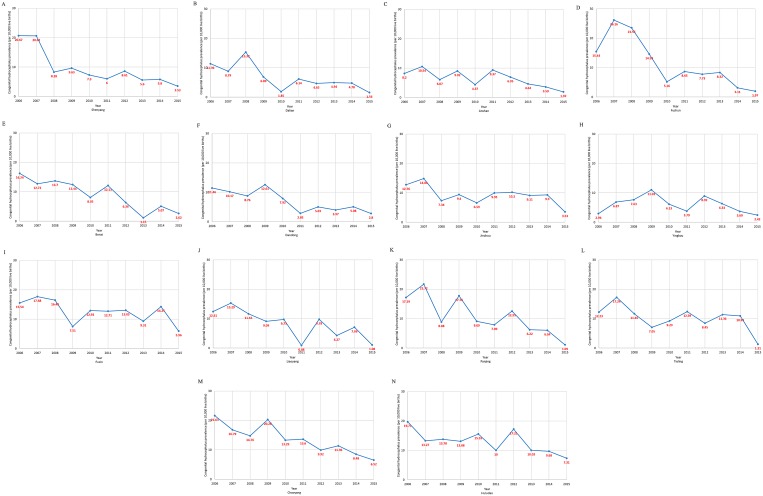
Trends in congenital hydrocephalus prevalence (per 10,000 live births) in 14 cities in Liaoning province from 2006 to 2015 on the basis of a population-based birth defect registry from Liaoning Women and Children's Health Hospital (**A**) Shenyang; (**B**) Dalian; (**C**) Anshan; (**D**) Fushun; (**E**) Benxi; (**F**) Dandong; (**G**) Jinzhou; (**H**) Yingkou; (**I**) Fuxin; (**J**) Liaoyang; (**K**) Panjing; (**L**) Tieling; (**M**) Chaoyang; (**N**) Huludao.

The prevalence of CH in Liaoning province from 2006 to 2015 is presented in Figure 3. The overall prevalence significantly decreased from 14.64 to 3.18 cases per 10,000 live births, or 11.84% per year (*p* < 0.01). Evaluation of the trend in CH prevalence demonstrated a decreasing trend in all cities of Liaoning province with a significant decrease in 10 (71.4%) of the 14 cities (Table 2 and Figure 4). Notably, the cities of Fushun, Shenyang, and Dalian contributed to more than one-third of the decreasing trend in the prevalence of CH in Liaoning province (Table 3).

**Table 2:** Trends in CH prevalence in each city of Liaoning during 2006-2015

CH	2006		2015		PC^†^ (%)	AC^†^ (%)	*P* value	95% CI
	Case	Rate^*^	Case	Rate^*^				
Overall	449	14.64	95	3.18	−78.25	−11.84	< 0.01	−15.03, −8.52
Shenyang	108	20.67	23	3.53	−82.91	−16.47	< 0.01	−22.06, −10.49
Dalian	44	11.36	9	1.56	−86.25	−14.53	0.01	−23.31, −4.75
Anshan	24	8.20	4	1.92	−76.54	−9.34	0.04	−17.13, −0.80
Fushun	18	15.44	2	1.97	−87.22	−19.27	< 0.01	−28.06, −9.40
Benxi	14	16.24	2	2.62	−83.85	−14.52	< 0.01	−21.70, −6.70
Dandong	18	11.46	4	2.80	−75.55	−12.98	< 0.01	−19.35, −6.10
Jinzhou	31	12.76	6	3.53	−72.32	−6.29	0.07	−12.76, 0.65
Yingkou	5	2.94	4	2.42	−17.71	−4.78	0.42	−16.70, 8.84
Fuxin	22	15.54	7	5.96	−61.67	−5.92	0.07	−12.00, 0.59
Liaoyang	16	12.41	1	1.08	−91.29	−11.04	0.03	−19.62, −1.54
Panjing	17	17.19	1	1.09	−93.68	−13.50	0.01	−22.20, −3.82
Tieling	26	12.23	2	1.31	−89.29	−5.45	0.18	−13.58, 3.45
Chaoyang	62	21.63	17	6.52	−69.86	−10.24	< 0.01	−14.09, −6.22
Huludao	44	19.71	13	7.31	−62.91	−6.01	0.04	−11.48, −0.20

AC, average change; CI, confidence interval; N/A, not available; PC, percent change.

^*^ CH prevalence is expressed as the number of cases per 10,000 live births.

^†^ The percent change and average change in CH prevalence between 2006 and 2015 were calculated

**Table 3:** The relative contributions of decreasing trend of CH prevalence of each city in Liaoning province during 2006–2015

City	Decreasing trend	
	β	Contribution rate (%)
Shenyang	−0.18	11.19
Dalian	−0.16	9.76
Anshan	−0.10	6.09
Fushun	−0.21	13.31
Benxi	−0.16	9.75
Dandong	−0.14	8.64
Jinzhou	−0.07	4.04
Yingkou	−0.05	3.05
Fuxin	−0.06	3.79
Liaoyang	−0.12	7.28
Panjing	−0.15	9.02
Tieling	−0.06	3.48
Chaoyang	−0.11	6.72
Huludao	−0.06	3.86

## DISCUSSION

To the best of our knowledge, the present study is one of the few population-based reports of the prevalence of CH in China over the most recent decade. From 2006 to 2015, the prevalence of CH of Liaoning province significantly decreased from 14.64 to 3.18 cases per 10,000 live births due to decreasing trends observed in all 14 cities, although this trend did not reach statistical significance in all cities. Since the prevalence of CH remained relatively high in some cities, further preventive efforts are warranted to reduce the future risk of CH in these areas.

The results of the present study showed that the overall prevalence of CH in Liaoning province from 2006 to 2015 was 9.26 cases per 10,000 live births. Although geographic variation in the prevalence of this defect exists in different areas of different countries, compared to the results from recent studies performed in developed countries, these findings fall within the reported ranges of 2.5–10.4 cases per 10,000 live births [9–11, 13, 17, 19, 22]. In addition, a decreasing trend in the prevalence of CH was observed in these studies. Possible etiologies for this decline include implementation of folic acid supplementation, improved care of extremely preterm infants, and improved prenatal diagnosis leading to pregnancy termination [23]. Canada and the United States were the first countries to require mandatory fortification of enriched cereal grain products with 140 μg of folic acid per 100 g. Consequently, many studies have documented a reduction in these anomalies after implementation of this policy [24]. By mid-2012, a total of 67 counties required folic acid fortification of wheat flour (mandatory or voluntary programs), which affected approximately 2.2 billion people [24, 25]. However, fortification is uncommon in Asian and European countries, which may account for the regional differences in the prevalence of CH [26]. China initiated a nationwide folic acid supplementation program in 2009, which provides folic acid supplements, free of charge, to all women living in rural areas who plan to become pregnant [27]. The results of the present study showed that the prevalence of CH in 2012 to 2015 decreased dramatically from that of 2009 in the majority of the 14 surveyed cities, which may be partly attributable to the effects of this national policy. However, there was no significant decrease in the prevalence of CH in all 14 cities, suggesting that this phenomenon might be attributed to differences in the development of these cities and fewer women living in rural areas, as opposed to urban areas, of northern China had taken folic acid supplements before pregnancy [28]. Therefore, policymakers should pay more attention to this issue in these cities.

There were several strengths to this study. For example, the study data were collected from a population-based birth defect registry with good quality control [29] that included all 14 cities of Liaoning province over a relatively long time period (10 years) as well as the most recent data up to 2015, which comprehensively described the time trend and prevalence of CH in one of the most important provinces in China. However, this study is not without limitations. Firstly, when compared to the mature registry systems in developed countries, the Shenyang Women and Children Health Care Center began collecting information in 1992 and were inevitable to several common technical problems that could jeopardize the quality of the collected data [31], which might be responsible for under-reporting issue. Secondly, information pertaining to demographic factors of all live births in Liaoning province was not available, which hindered our ability to identify potential causes of the trends in the prevalence of CH. Furthermore, since the access on the data, we hardly analyzed distributions of CH prevalence by district/region of these cities. Thirdly, since the discharge diagnostic criteria of CH may differ between hospitals and individual coders, it is possible that these findings may be biased. Furthermore, it is difficult to know the degree to which this variation may be associated with other sources of variation [23]. Fourthly, the denominator of the prevalence of CH was the total number of births (live births and still births at a minimum of gestational week 28). Although the inclusion of induced and spontaneous fetal deaths before gestational week 28 would more closely approximate the prevalence of CH, it is very impractical, as these pregnancy outcomes are often inaccurately counted, as compared to live births and stillbirths [30]. In addition, as compared to the number of live births and stillbirths, the number of induced and spontaneous fetal deaths was small and thus unlikely to have greatly impacted the results [30]. Lastly, the maximal diagnosis time for CH cases was postnatal day 7 [29]. Hence, CH cases confirmed after postnatal day 7 were excluded from this study (*n* = 95). Therefore, the calculated prevalence of CH may be slightly lower than that over a longer period.

In summary, this population-based study provides recent and detailed evidence of the prevalence of CH between 2006 and 2015 in one of the most populated provinces in China. More importantly, the prevalence of CH remains high in some cities, which might draws attention to the need to improve the efficiency of periconceptional folic acid supplementation. Preventative efforts should be strengthened to reduce the risk of CH in areas with high prevalence.

## MATERIALS AND METHODS

### Study population and data source

Liaoning Women and Children's Health Hospital is a primary obstetrical and gynecological hospitals as well as an official organization of government in Liaoning province that offers comprehensive healthcare to women and children. Data from 2006 to 2015 were retrieved from the maternal and child health certificate registry of Liaoning province maintained by this hospital. Hospital-delivered live birth and stillbirth infants in Liaoning province were all included in this registry as monitored subjects. The registry covers all 14 cities in the province (Shenyang, Dalian, Anshan, Fushun, Benxi, Dandong, Jinzhou, Yingkou, Fuxin, Liaoyang, Panjing, Tieling, Chaoyang, and Huludao), with approximately 42 million inhabitants. The maximal diagnosis time for a congenital malformation case was postnatal day 7 [29]. One hundred and nineteen city and counties institutions of maternal and child health in Liaoning province monthly uploaded the data to the Liaoning Women and Children's Health Hospital.

The detailed procedures of data collection are described in a previous report [29]. Briefly, a Birth Defect Registry Form was used to collect relevant information about infants with CH. We defined CH according to the World Health Organization's International Classification of Diseases, 10th Revision. Once a CH case was identified and confirmed at the monitored hospital, the mother of the infant was interviewed by a trained obstetric or pediatric specialist in order to complete the aforementioned registry form. Subsequently, the form was first submitted to the local maternal and child healthcare facility and then to the provincial maternal and child healthcare hospital, which was Liaoning Women and Children's Health Hospital. The data of these cases were reviewed and confirmed by a group of state-level experts in medical genetics and pediatrics [29].

All cases of CH were included for analysis. The prevalence of CH at birth is presented as the number of cases per 10,000 live births. The denominator was based exclusively on the total number of live births and data were obtained primarily from the database of Liaoning Women and Children's Health Hospital. For suspected cases of CH that were diagnosed through prenatal ultrasound scans, cases were ascertained after termination or postnatal examination. During the 10-year study period (2006–2015), a total of 3008 cases of CH were identified among a total of 3, 248, 954 live births.

Quality control of the data is described in detail elsewhere [29]. In brief, to ensure high quality data, disease diagnosis, data collection, data checking, and medical records were verified by an expert group at each level. In addition, an independent retrospective survey was conducted by experts to identify deficiencies and inaccuracies in the data [29]. This study protocol was approved by the Institutional Review Board of Liaoning Women and Children's Health Hospital and conducted in compliance with local and national regulations.

### Statistical analysis

The prevalence of CH was calculated for ten 1-year time intervals from 2006 to 2015. In order to specifically assess time trends, a Poisson regression model was used to identify a line of best fit for the prevalence of CH by year, with year entered into the model as a continuous independent variable. The detail method of 95% confidence interval (CI) of the average change was described in previous report [31, 32]. Relative contributions to rate changes were calculated to provide the contributions of each city to the overall trend [31–33]. All analyses were conducted using IBM SPSS Statistics for Windows, version 23.0 (IBM Corp., Armonk, NY, USA). All statistical tests were two-sided and a probability (*P*) value of < 0.05 was considered statistically significant.

## References

[1] Aicardi J, Bax M, Ogier H, Gillberg C (1998). Diseases of the nervous system in childhood.

[2] Wu Y, Green NL, Wrensch MR, Zhao S, Gupta N (2007). Ventriculoperitoneal shunt complications in California: 1990 to 2000. Neurosurgery.

[3] Partington MD (2001). Congenital hydrocephalus. Neurosurg Clin N Am.

[4] Van Landingham M, Nguyen TV, Roberts A, Parent AD, Zhang J (2009). Risk factors of congenital hydrocephalus: a 10 year retrospective study. J Neurol Neurosurg Psychiatry.

[5] Graff-Radford NR, Godersky JC (1989). Symptomatic congenital hydrocephalus in the elderly simulating normal pressure hydrocephalus. Neurology.

[6] Krefft TA, Graff-Radford NR, Lucas JA, Mortimer JA (2004). Normal pressure hydrocephalus and large head size. Alzheimer Dis Assoc Disord.

[7] Wilson RK, Williams MA (2007). Evidence that congenital hydrocephalus is a precursor to idiopathic normal pressure hydrocephalus in only a subset of patients. J Neurol Neurosurg Psychiatry.

[8] Dai L, Zhou GX, Miao L, Zhu J, Wang YP, Liang J (2006). [Prevalence analysis on congenital hydrocephalus in Chinese perinatal from 1996 to 2004]. Zhonghua Yu Fang Yi Xue Za Zhi.

[9] Garne E, Loane M, Addor MC, Boyd PA, Barisic I, Dolk H (2010). Congenital hydrocephalus--prevalence, prenatal diagnosis and outcome of pregnancy in four European regions. Eur J Paediatr Neurol.

[10] Persson EK, Anderson S, Wiklund LM, Uvebrant P (2007). Hydrocephalus in children born in 1999–2002: epidemiology, outcome and ophthalmological findings. Childs Nerv Syst.

[11] Stoll C, Alembik Y, Dott B, Roth MP (1992). An epidemiologic study of environmental and genetic factors in congenital hydrocephalus. Eur J Epidemiol.

[12] Murshid WR, Jarallah JS, Dad MI (2000). Epidemiology of infantile hydrocephalus in Saudi Arabia: birth prevalence and associated factors. Pediatr Neurosurg.

[13] Forrester MB, Merz RD (2005). Descriptive epidemiology of congenital hydrocephaly in Hawaii, 1986--2000. Hawaii Med J.

[14] Fernell E, Hagberg B, Hagberg G, von Wendt L (1987). Epidemiology of infantile hydrocephalus in Sweden. II. Origin in infants born at term. Acta Paediatr Scand.

[15] Fernell E, Hagberg B, Hagberg G, von Wendt L (1987). Epidemiology of infantile hydrocephalus in Sweden. III. Origin in preterm infants. Acta Paediatr Scand.

[16] Fernell E, Hagberg B, Hagberg G, von Wendt L (1986). Epidemiology of infantile hydrocephalus in Sweden. I. Birth prevalence and general data. Acta Paediatr Scand.

[17] Sipek A, Gregor V, Horacek J, Masatova D (2002). [Congenital hydrocephalus 1961–2000--incidence, prenatal diagnosis and prevalence based on maternal age]. Ceska Gynekol.

[18] Fernell E, Hagberg G (1998). Infantile hydrocephalus: declining prevalence in preterm infants. Acta Paediatr.

[19] Glinianaia SV, Rankin J (1999). Congenital hydrocephalus: occurrence and outcome. A population-based study in the North of England, 1985–1996. Northern Congenital Abnormality Survey Steering Group. Eur J Pediatr Surg.

[20] Liu S, Joseph KS, Kramer MS, Allen AC, Sauve R, Rusen ID, Wen SW (2002). Relationship of prenatal diagnosis and pregnancy termination to overall infant mortality in Canada. JAMA.

[21] Wang YP, Liang J, Zhu J, Wu YQ, Miao L, Zhou GX, Dai L (2000). The dynamic variation of prevalence of congenital hydrocephaly over the year 1988–1992 in China. Journal of Applied Clinical Pediatrics.

[22] Fernell E, Hagberg G, Hagberg B (1990). Infantile hydrocephalus--the impact of enhanced preterm survival. Acta Paediatr Scand.

[23] Jeng S, Gupta N, Wrensch M, Zhao S, Wu YW (2011). Prevalence of congenital hydrocephalus in California, 1991–2000. Pediatr Neurol.

[24] Atta CA, Fiest KM, Frolkis AD, Jette N, Pringsheim T, St Germaine-Smith C, Rajapakse T, Kaplan GG, Metcalfe A (2016). Global Birth Prevalence of Spina Bifida by Folic Acid Fortification Status: A Systematic Review and Meta-Analysis. Am J Public Health.

[25] Food Fortification Initiative Enhancing Grains for Healthier Lives. Country profiles. http://www.ffinetwork.org/country_profiles/index.php.

[26] Zaganjor I, Sekkarie A, Tsang BL, Williams J, Razzaghi H, Mulinare J, Sniezek JE, Cannon MJ, Rosenthal J (2016). Describing the Prevalence of Neural Tube Defects Worldwide: A Systematic Literature Review. Plos One.

[27] Ren AG (2015). Prevention of neural tube defects with folic acid: The Chinese experience. World J Clin Pediatr.

[28] Liu J, Jin L, Meng Q, Gao L, Zhang L, Li Z, Ren A (2015). Changes in folic acid supplementation behaviour among women of reproductive age after the implementation of a massive supplementation programme in China. Public Health Nutr.

[29] Xu L, Li X, Dai L, Yuan X, Liang J, Zhou G, Li Q, He C, Miao L, Wang Y, Zhu J (2011). Assessing the trend of gastroschisis prevalence in China from 1996 to 2007 using two analytical methods. Birth Defects Res A Clin Mol Teratol.

[30] Li X, Zhu J, Wang Y, Mu D, Dai L, Zhou G, Li Q, Wang H, Li M, Liang J (2013). Geographic and urban-rural disparities in the total prevalence of neural tube defects and their subtypes during 2006–2008 in China: a study using the hospital-based birth defects surveillance system. Bmc Public Health.

[31] Wu QJ, Li LL, Li J, Zhou C, Huang YH (2016). Time trends of neonatal mortality by causes of death in Shenyang, 1997–2014. Oncotarget.

[32] Huang YH, Wu QJ, Li LL, Li D, Li J, Zhou C, Wu L, Zhu J, Gong TT (2016). Different extent in decline of infant mortality by region and cause in Shenyang, China. Sci Rep.

[33] Wu QJ, Vogtmann E, Zhang W, Xie L, Yang WS, Tan YT, Gao J, Xiang YB (2012). Cancer incidence among adolescents and young adults in urban Shanghai, 1973–2005. Plos One.

